# Complications of Biliary Fistula with Pregnancy

**Published:** 1888-02

**Authors:** 


### Selections.

---

Complication of Biliary Fistula with Pregnancy.—*Case* 1. In September, 1885, I was called to see Mrs. H., aged thirty-five years, the mother of eight children, suffering with hepatic colic.

I soon discovered that I had a case of impacted gall-stone, as within a few days a tumor began to appear at the lower border of the liver. After using various measures, and no relief, I advised cholecystotomy to relieve the urgent symptoms, but they would not agree to it. The case finally drifted into the hands of another party, who proposed to get rid of the tumor by "scattering" it.

I did not hear from the case for six months, when the husband informed me that the tumor had ruptured through the abdominal walls, discharging eleven gall-stones. I continued to hear from the case occasionally until July, 1887, when she sent for me to perform the operation.

I went to see her and found that she was five months pregnant. I had thought that it might be best to enlarge the fistula and explore the gall-bladder, but when she informed me that she was pregnant, I refused to perform any operation, for fear of bringing on premature labor, and thus rupturing the adhesions between the gall-bladder and the abdominal walls.

The case ran on until the 16th of November, when she was safely delivered of a healthy child. For four weeks before her confinement, the side was enormously swollen from the lower border of the breast to the crest of the ilium.

It was apparent that something must give way, and on the 13th, before her confinement, the sac ruptured into the bowel, when in a few minutes afterwards she vomited six or eight ounces of mucus and bile. In two hours she was up and around the room, feeling very much relieved.

When labor first commenced, there was probably a drachm of bile squeezed out through the fistula, but during the latter stage of labor there was none whatever. I had feared that the descent of the diaphragm would rupture the adhesions, but only once she complained of pain in the hepatic region.

So far as I could detect, the diaphragm and abdominal muscles took no part, or but very little, in the labor.

I am unable to give more than a conjecture what part of the alimentary tract the distended gall-bladder ruptured into. Whether the upward pressure forced the impacted stone out through the common duct, or if during the acute symptoms the gall-bladder became adherent to some part of the duodenum, and was discharged in that way.

In October I wrote Dr. Gaston, of Atlanta, asking his opinion of the probable termination of the case. He answered "that he had not met with a like case, but that the upward pressure of the gravid uterus may force out any stones or other collection in the gall-bladder." His suppositions were verified in this case, and I shall hope that the doctor may always be as accurate in his prognosis.

At this date the fistula has about healed, the opening being very small. If it does not close within a short time, I shall pass a probe, hoping to set up sufficient inflammation to cause the sinus to granulate. Owing to the improvement in her general health, and the subsidence of all inflammation, I am satisfied that the contents of the gall-bladder are passing into the bowel.

The adhesions between the parts were very extensive, with a large amount of thickening and induration, and it may be quite a while before absorption takes place, if it ever does entirely.

In the next case of biliary tumor that presents itself (if I am unable to recommend pregnancy) I shall perform Gaston's operation—stitching the gall-bladder to the duodenum. I am convinced that the operation is feasible, and would be much safer than the running of a trocar through the common duct. I am aware that the weight of authority says to let a biliary fistula alone, but if the adhesions were not too extensive, and if not of oo long standing, I should recommend the operation to relieve one of the most distressing conditions that human kind are subject to.—*J. W. Long, M. D., Russell, Kansas, in Weekly Medical Review.*

